# Transfer Learning for Toxoplasma gondii Recognition

**DOI:** 10.1128/mSystems.00445-19

**Published:** 2020-01-28

**Authors:** Sen Li, Aijia Li, Diego Alejandro Molina Lara, Jorge Enrique Gómez Marín, Mario Juhas, Yang Zhang

**Affiliations:** a College of Science, Harbin Institute of Technology, Shenzhen, China; b GEPAMOL Group, Faculty of Health Sciences, University of Quindío, Armenia, Colombia; c Institute of Medical Microbiology, University of Zürich, Zürich, Switzerland; University of Michigan-Ann Arbor

**Keywords:** *Toxoplasma gondii*, artificial intelligence, transfer learning, microscopic image, banana shaped

## Abstract

Toxoplasma gondii, one of the world’s most common parasites, can infect all types of warm-blooded animals, including one-third of the world’s human population. Most current routine diagnostic methods are costly, time-consuming, and labor-intensive. Although T. gondii can be directly observed under the microscope in tissue or spinal fluid samples, this form of identification is difficult and requires well-trained professionals. Nevertheless, the traditional identification of parasites under the microscope is still performed by a large number of laboratories. Novel, efficient, and reliable methods of T. gondii identification are therefore needed, particularly in developing countries. To this end, we developed a novel transfer learning-based microscopic image recognition method for T. gondii identification. This approach employs the fuzzy cycle generative adversarial network (FCGAN) with transfer learning utilizing knowledge gained by parasitologists that *Toxoplasma* is banana or crescent shaped. Our approach aims to build connections between microscopic and macroscopic associated objects by embedding the fuzzy C-means cluster algorithm into the cycle generative adversarial network (Cycle GAN). Our approach achieves 93.1% and 94.0% detection accuracy for ×400 and ×1,000 *Toxoplasma* microscopic images, respectively. We showed the high accuracy and effectiveness of our approach in newly collected unlabeled *Toxoplasma* microscopic images, compared to other currently available deep learning methods. This novel method for *Toxoplasma* microscopic image recognition will open a new window for developing cost-effective and scalable deep learning-based diagnostic solutions, potentially enabling broader clinical access in developing countries.

**IMPORTANCE** Toxoplasma gondii, one of the world’s most common parasites, can infect all types of warm-blooded animals, including one-third of the world’s human population. Artificial intelligence (AI) could provide accurate and rapid diagnosis in fighting *Toxoplasma*. So far, none of the previously reported deep learning methods have attempted to explore the advantages of transfer learning for *Toxoplasma* detection. The knowledge from parasitologists is that the *Toxoplasma* parasite is generally banana or crescent shaped. Based on this, we built connections between microscopic and macroscopic associated objects by embedding the fuzzy C-means cluster algorithm into the cycle generative adversarial network (Cycle GAN). Our approach achieves high accuracy and effectiveness in ×400 and ×1,000 *Toxoplasma* microscopic images.

## Abstract (precis)

Toxoplasma gondii, one of the world’s most common parasites, can infect all types of warm-blooded animals, including one-third of the world’s human population. Artificial intelligence (AI) could provide accurate and rapid diagnosis in fighting *Toxoplasma*. So far, none of the previously reported deep learning methods have attempted to explore the advantages of transfer learning for *Toxoplasma* detection. The knowledge from parasitologists is that the *Toxoplasma* parasite is generally banana or crescent shaped. Based on this, we built connections between microscopic and macroscopic associated objects by embedding the fuzzy C-means cluster algorithm into the cycle generative adversarial network (Cycle GAN). Our approach achieves high accuracy and effectiveness in ×400 and ×1,000 *Toxoplasma* microscopic images.

## INTRODUCTION

**FIG 1:**
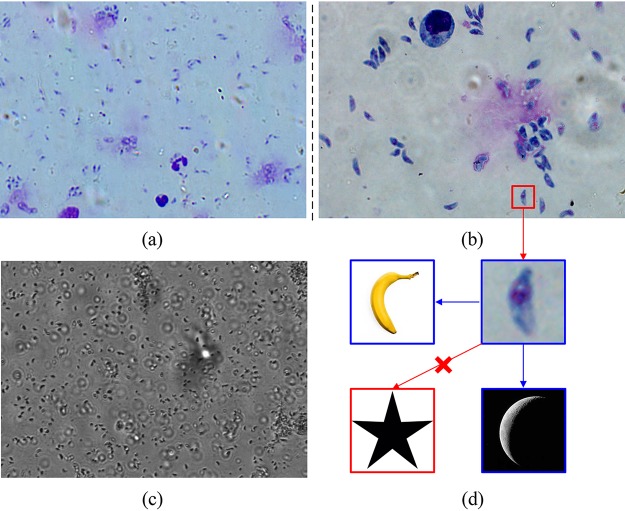
Typical variations of color, illumination, and background in different fields of view and microscope magnifications. (a) Stained T. gondii images from different slides captured by a microscope with ×400 magnification. (b) Stained T. gondii images from different slides captured by a microscope with ×1,000 magnification. (c) Unstained T. gondii image captured by a microscope with ×400 magnification. (d) Training image data with similar macroscopic object sources (banana and crescent) and a different macroscopic object (star).

Toxoplasma gondii is a ubiquitous, single-cell protozoan parasite that can infect all warm-blooded animals as well as one-third of the human population worldwide (1). Most infections in humans are lifelong, and several studies have suggested that such infections may contribute to severe neurological and psychiatric symptoms. That makes diseases caused by T. gondii one of the biggest health care problems globally. Diagnosis of T. gondii is typically performed by testing blood or other body fluids for antibodies or the parasite’s DNA (2). Large numbers of laboratories still perform identification of T. gondii in tissue or spinal fluid samples under the microscope. However, the microscopic detection and quantification of T. gondii are time-consuming and labor-intensive and need well-trained professionals. Moreover, the intensity of illumination, image brightness, contrast level, and background of staining have large variations in different fields of view (FoV). In addition, T. gondii is found both inside and outside nucleated cells with separated and aggregated forms, which makes the task of T. gondii recognition more challenging (Fig. 1).

Recently, deep learning technology has significantly improved efficiency and accuracy in macroscopic computer vision tasks, thereby attracting considerable attention in microscopic image analysis (3–6). Rajaraman et al. (7) evaluated the performance of a pretrained convolutional neural network (CNN) as a feature extractor in the classification of parasites and host cells, which improved infectious disease screening. Furthermore, Mehanian et al. (8) developed a computer vision system with deep learning to identify malaria parasites under the microscope in field-prepared thick blood films. However, most of the existing deep learning methods for parasite analysis are under a supervised learning framework, which requires many well-trained professionals to label a number of image data sets. Furthermore, labeling, annotating, and sorting the output data are time-consuming, costly, and labor-intensive. This severely limits their scalability in practical applications.

Moreover, current existing parasite recognition models are limited mainly to malaria. Here, we proposed a deep learning method for T. gondii recognition. To improve learning efficiency in the task, we employed a transfer learning strategy by leveraging knowledge from parasitologists that T. gondii is crescent shaped, similar to a banana. Furthermore, the shape of aggregated parasites resembles the images of a bunch of bananas, where host cells are significantly different. It is assumed that a microscopic object has an inherent connection with the macroscopic world. Based on this assumption, we designed a microscopic-macroscopic associated object pulling (M2AOP) strategy and proposed a fuzzy cycle generative adversarial network (FCGAN) for T. gondii recognition. This strategy embeds a fuzzy C-means (FCM) cluster algorithm (9) into the cycle generative adversarial network (Cycle GAN) (10), which can learn degrees of membership belonging to each cluster (class) point. Then, the degrees are utilized as the translated coefficient to replace the microscopic and macroscopic domain labels in Cycle GAN when the microscopic images are inductively transferred into the macroscopic world. FCGAN can exploit more discriminative information using a third associated object to asymmetrically pull T. gondii and host cell samples. Prior knowledge on the features selected is not necessary, avoiding bias caused by hand-picking. Transferring knowledge from the macroscopic domains can eliminate interference in microscopic domains while retaining features crucial to identifying microscopic objects.

We tested two T. gondii image data sets consisting in total of 13,135 microscopic images with a magnification of ×400 (T400) and 14,992 microscopic images with a magnification of ×1,000 (T1000). A number of experiments were conducted demonstrating the effectiveness of our FCGAN method with high accuracy and precision. Using macroscopic knowledge to guide our model, the present study is the first of its kind to investigate the utility of a deep learning algorithm for T. gondii parasite recognition. To our knowledge, based on thorough investigation of the existing literature, no previous research has explored the potential of transferring human knowledge for T. gondii parasite recognition.

## RESULTS

### Recognition performance of the model.

As shown in Table 1, for recognition performance in ×400 and ×1,000 T. gondii data sets, our FCGAN with banana source data achieves the best performance in all unsupervised models with small difference from supervised deep learning models (ResNet, VggNet, and GoogleNet). For comparison with supervised methods, we selected three widely used supervised CNNs including VggNet, GoogleNet, and ResNet. As shown in Table 1, it can be seen that ResNet, GoogleNet, and VggNet obtain 98.4%, 98.7%, and 97.2% accuracy in the T400 data set, respectively. FCGAN with banana source achieves a competitive result of 93.1% accuracy, 0.939 F1 score, 0.960 recall, and 0.919 precision on the T400 data set and 94.0% accuracy, 0.939 F1 score, 0.929 recall, and 0.949 precision on the T1000 data set. The values of accuracy, F1 score, precision, and recall in our model are similar to those from ResNet, VggNet, and GoogleNet. All those results demonstrate that our FCGAN can achieve similar performance as supervised deep learning methods, while FCGAN does not require labeling of any microscopic images. It should be emphasized that T. gondii labeling under the microscope is time-consuming and labor-intensive and requires well-trained professionals.

**TABLE 1:** Results of FCGAN and baselines on T400 and T1000 *Toxoplasma* data sets^a^

Model	Parameter for data set:							
	T400				T1000			
	% accuracy	F1 score	Recall	Precision	% accuracy	F1 score	Recall	Precision
Supervised learning method								
ResNet	98.4	0.986	0.982	0.990	98.9	0.989	0.984	0.995
VggNet	98.7	0.989	0.996	0.982	99.1	0.991	0.990	0.991
GoogleNet	97.2	0.974	0.950	0.998	99.6	0.997	0.999	0.993
Unsupervised learning method								
ResFCM	84.3	0.851	0.817	0.889	80.0	0.801	0.803	0.800
VggFCM	77.4	0.760	0.650	0.916	87.6	0.863	0.785	0.960
GoogleFCM	74.3	0.758	0.730	0.789	81.5	0.797	0.728	0.880
CycleFCM	86.9	0.889	0.955	0.832	86.2	0.862	0.861	0.863
FuzzyFCM	86.0	0.887	0.992	0.802	91.7	0.918	0.933	0.904
GANFCM	81.0	0.838	0.890	0.792	88.9	0.892	0.911	0.873
**FCGAN+Star**	74.8	0.767	0.749	0.786	87.0	0.867	0.845	0.889
**FCGAN+Crescent**	92.0	0.927	0.916	0.938	92.3	0.926	0.950	0.903
**FCGAN+Banana**	**93.1**	**0.939**	**0.960**	**0.919**	**94.0**	**0.939**	**0.929**	**0.949**

a Our FCGAN method with the guidance of different macroscopic source objects are highlighted in boldface.

**FIG 2:**
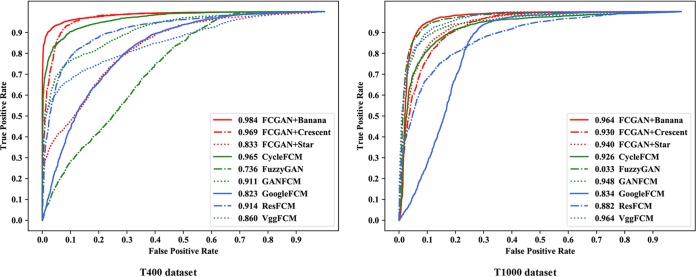
ROC curves of different methods for *Toxoplasma* recognition in T400 and T1000 data sets. The AUC value is given for each method. FCGAN with banana source achieves the best performance compared to other unsupervised methods.

In comparison with unsupervised methods, we tested five unsupervised baselines together with our FCGAN, including VggFCM, ResFCM, GoogleFCM, CycleFCM, and GanFCM. The results are illustrated in Table 1, and receiver operating characteristic (ROC) curves with area under the curve (AUC) values are drawn in Fig. 2. As shown, it is easy to prove that our FCGAN achieves the best performance compared to other unsupervised methods, outperforming the baselines at least 6.2% (93.1% to 86.9%) in accuracy, 0.05 (0.939 to 0.889) in F1 score, 0.005 (0.960 to 0.955) in recall, and 0.003 (0.919 to 0.916) in precision on the T400 data set.

To discuss the effectiveness of source object, we then compared the results from banana, crescent, and star images as sources. In morphology, a star is less similar to T. gondii than banana and crescent, and its results and ROC curves have a distance between FCGAN+Banana and FCGAN+Crescent in Table 1 and Fig. 2. The accuracy results are 93.1% (94.0%) of banana source, 92.0% (92.3%) of crescent source, and 74.8% (87.0%) of star source on the T400 (T1000) data set. It can be seen that FCGAN+Banana obtains a better performance than FCGAN+Crescent and FCGAN+Star in both data sets. Those results indicate that, the more similar the macroscopic object, the better the performance achieved in our FCGAN model.

Taken together, our microscopic-macroscopic associated object pulling strategy with banana source can achieve competitive results for T. gondii recognition without any microscopic image labels.

### Evaluation of the fuzzy component.

**FIG 3:**
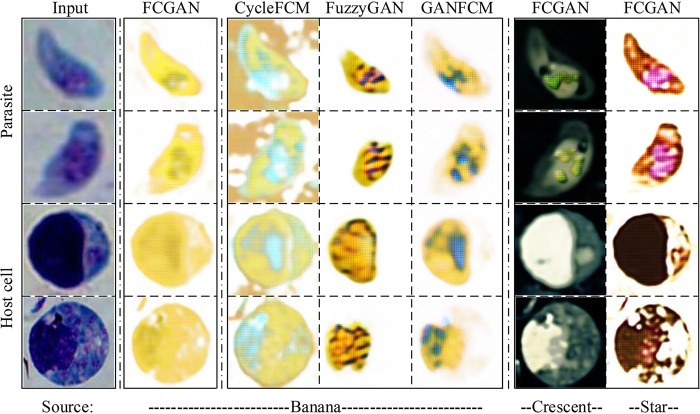
Image translation generated from different sources by FCGAN and other GAN-based methods on *Toxoplasma* cells and host cells. Compared to the GAN-based methods, the images generated from FCGAN contain more texture and shape details. Most notably, FCGAN with banana source has learned to reconstruct the nucleus of T. gondii.

In Fig. 3, we illustrate a few samples of the translated images generated from FCGAN with different source objects and GAN-based baselines. FCGAN preserves most information in translated images, including shape, nucleus, and texture information, with the same background and color as their source objects. But, for GAN-based methods, CycleFCM keeps the most background information for images, and FuzzyGAN generates target objects only partially, omitting some morphology information for both T. gondii and the host cell. Similarly, GAN+FCM leaves out some morphology features for the host cell. Besides, compared with different source objects, FCGAN with banana source produces a clear outline without any background, compared to other source objects.

Together, our FCGAN can create the texture and shape relationship between microscopic object and macroscopic object with selectivity of associated information. The comparison of different models can show that our FCGAN with banana source can achieve a better performance, indicating that FCGAN can exploit associated information (for example, shape and texture information) to boost the cluster results.

### Analysis of network layers.

**FIG 4:**
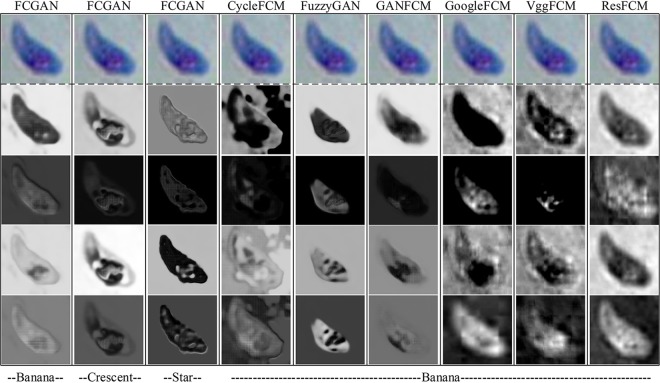
Visualization of the convolutional feature map learned by FCGAN and baselines on *Toxoplasma* cell. Object outline has been extracted and visualized for the first convolutional layer of FCGAN and other baselines.

**FIG 5:**
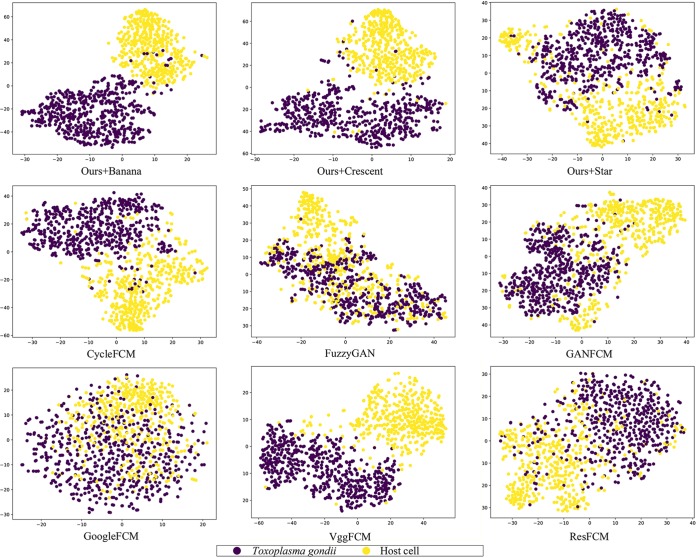
t-SNE plot of FCGAN and other baselines. t-SNE mappings provide a method to evaluate and refine clustering of parasite and cell images. Data points are colored according to cluster membership. The values on the *x* and *y* axes indicate the relative distance between the data points.

To analyze the network layers, we did feature map visualization for the first convolutional layer (Fig. 4). Feature visualization is a key technique that helps identify and recognize patterns inside neural networks. T-distributed stochastic neighbor embedding (t-SNE) plots for the last layer were performed for all models (Fig. 5). t-SNE is a nonlinear dimensionality reduction approach that allows embedding of high-dimensional data in a lower-dimensional space.

FCGAN can extract the outline of target images with clearer morphology than other baselines. FCGAN focuses on their texture information and produces clear feature maps for these data. FCGAN with banana source preserves the nucleus information in the T. gondii image. Nucleus information for T. gondii is lost with crescent and star sources. FCGAN achieves the best t-SNE plot performance compared to other baselines. The t-SNE plot shows that the feature extracted by the last layer in FCGAN with banana source is the best discriminated. All these results can prove that our FCGAN focuses on a clear outline of T. gondii in shallow convolutional layers and can extract discriminative information in the network.

### Application of the system for unstained T. gondii detection.

To investigate if our system can be used in the diagnosis of other T. gondii microscopic images, we applied the same transfer learning framework to analysis of unstained parasite images (Fig. 1c). Five hundred unstained T. gondii images and equal amount of unstained host cell images were collected to feed into our model. We achieved 90.3% accuracy, 0.910 F1 score, 0.986 recall, and 0.846 precision on this new unstained data set. This result shows that our FCGAN works not only on a large-scale stained parasite data set but also on an unstained one with a limited number of data.

Microscope staining is a key technique used to enable better visualization under the microscope. A variety of staining techniques can be used with light microscopy. Labeling and staining of parasites in microscopic images have always been challenging. As a result, an artificial intelligence (AI)-powered system for parasite recognition without any staining might open a new area for parasite detection showing potential to overcome the drawbacks of staining techniques.

## DISCUSSION

To our knowledge, this is the first deep learning study of the T. gondii recognition task and transfer learning-based fuzzy cycle generative adversarial network approach. The model is inspired by the knowledge from parasitologists that T. gondii is banana or crescent shaped.

In this context, resemblances between macroscopic images and microscopic images are exploited in order to train the data set. We designed a microscopic-macroscopic associated object pulling strategy and proposed the FCGAN method for T. gondii microscopic image recognition without any data annotation. This method uses the fuzzy clustering algorithm to improve selectivity and optimize cycle consistency and associated object pulling losses.

Using T. gondii microscopic image data sets with magnifications of ×400 and ×1,000, we successfully demonstrated that our FCGAN model has stronger associated information selectivity and better pulling effect than the other deep learning approaches. We employed different source object data with differing similarities to T. gondii. The ranking of performance in different source objects is banana > crescent > star, which is consistent with their similarity to *Toxoplasma* in shape.

To demonstrate the general applicability of our system, we tested T. gondii images captured by different equipment. The stained T. gondii microscopic images were collected in China; among them ×400 images were captured by the Leica microscope and ×1,000 images were captured by the Olympus microscope, which achieved accuracies of 93.1% and 94%, respectively. The unstained images were collected in Colombia with ×400 magnification by the EVOS FL Auto cell imaging system, achieving 90.3% accuracy. That illustrates that our FCGAN method can cope with various data from different equipment.

Our method achieves similar classification accuracy when separating T. gondii from host cells without using labeled *Toxoplasma* images to train the model but instead using banana, crescent, and star images. The proposed technique, while obtaining worse results than established supervised techniques, outperforms unsupervised techniques by using an “analogy” macroscopic data set instead of a real microscopic data set of T. gondii.

However, the performance of our model depends highly on the macroscopic object images resembling the objects in the microscopic images. Therefore, the performance of this model would likely be enhanced by testing on a more-similar source image data set. If this approach needs to be applied to another biomedical image domain, a macroscopic object that best matches the microscopic objects of interest is required.

In addition, the variation present in macroscopic object photos is significantly influenced by the fact that they are two-dimensional (2D) views of three-dimensional (3D) objects whereas cells and parasites are flattened 3D objects with the sharpest-focus image acquired. Therefore, multiple microscopic images taken at different focus distances need to be tested in the future.

Some other domain knowledge can be combined in the deep learning framework, such as appearance, geometric characteristics, or statistical properties. However, it is unclear which knowledge is necessary to improve prediction. Therefore, this paper proposes a method transferring knowledge between microscopic and macroscopic domains. As a convolutional neural network (CNN) can learn the features necessary for classification, prior knowledge on the features that are beneficial for T. gondii recognition is not necessary. In future research, other domain knowledge such as shape, size, color, and statistical properties needs to be tested individually in order to highlight the most important features that have led to the high accuracy obtained in the present study. Then, this particular knowledge can be integrated into the deep learning framework to make a better and more reliable prediction.

Nevertheless, our method for *Toxoplasma* microscopic image analysis can potentially speed up detection and pave the way for rapid, low-cost diagnostics. Moreover, FCGAN can be applied to other biomedical image recognition tasks which have complicated procedures in data collection and annotation. Our model can learn useful knowledge from macroscopic related objects without time-consuming and labor-intensive image annotation and labeling.

## MATERIALS AND METHODS

**FIG 6:**
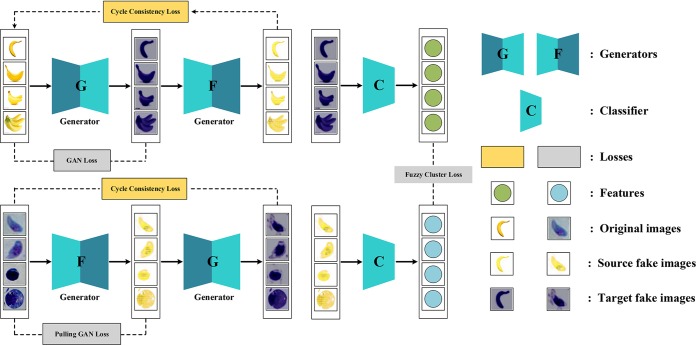
Schematic representation of FCGAN. Losses are shown in dashed rectangles. After training with source and target data alternatively, the generators *G* and *F* are used to pull T. gondii into the source domain and keep host cells in the target domain according to their shape and texture information. Discriminators *D_S_* and *D_T_* are not shown in the architecture. Source fake images are the output of target images through generator *F*, and target fake images are the output of source image through generator *G*. Features are extracted by a classifier *C* for both images from source and those from target.

Our proposed method is empowered by a microscopic-macroscopic associated object pulling strategy for microscopic parasite recognition. We utilized a fuzzy cycle generative adversarial network (FCGAN), shown in Fig. 6, where cycle consistency and fuzzy discriminator loss are optimized along with a fuzzy C-means algorithm.

### Backbone network design.

To extract robust features for microscopic images, we constructed a deep neural network to compute the representation of each sample *x* by passing it to multiple layers of nonlinear transformations. The key advantage of using such a network to map *x* is that the nonlinear mapping function can be explicitly obtained. There are *M *+ 1 layers in the designed network and *p*^(^*^m^*^)^ units in the *m*th layer, where *m* = 1, 2, …, *M*. The output of *x* at the *m*th layer is computed as:

(1) $$f^{\left(m\right)}(x)=h^{\left(m\right)}=\phi (W^{\left(m\right)}h^{\left(m-1\right)}+b^{\left(m\right)})\in {\mathbb{R}}^{p^{\left(m\right)}}$$

where $W^{\left(m\right)}\in {\mathbb{R}}^{p^{\left(m\right)}\times p^{\left(m-1\right)}}$ and $b^{\left(m\right)}\in {\mathbb{R}}^{p^{\left(m\right)}}$ are the weight matrix and bias of the parameters in this layer, respectively, and *ϕ* is a nonlinear activation function which operates component-wisely, such as widely used *tanh* or *sigmoid* functions. The nonlinear mapping $f^{\left(m\right)}:{\mathbb{R}}^{d}\to {\mathbb{R}}^{p^{\left(m\right)}}$ is a function parameterized by ${\left\{W^{\left(i\right)}\right\}}_{i=1}^{m}$ and ${\left\{b^{\left(i\right)}\right\}}_{i=1}^{m}$. For the first layer, we assumed *h*^(0)^ = *x* and *p*^(0)^ = *d*.

In this paper, we employed two typical convolutional networks as the base framework in FCGAN, including visual geometry group network (VggNet) (11) and cycle generative adversarial network (10).

The VggNet architecture was introduced by Simonyan and Zisserman in their 2014 paper (11). This network is characterized by its simplicity, using only 3 × 3 convolutional layers stacked on top of each other. Reducing volume size is handled by maximum pooling. Two fully connected layers, each with 4,096 nodes, are then followed by a softmax layer.

Cycle GAN is a generative adversarial network (GAN) that uses two generators and two discriminators. We call one generator *G* and have it convert images from the *X* domain to the *Y* domain. The other generator is called *F* and converts images from *Y* to *X*. Each generator has a corresponding discriminator, which attempts to discriminate its synthesized images from real ones. Along with two components to Cycle GAN objective functions, an adversarial loss and a cycle consistency loss are essential to getting good results. A detailed description can be found in reference 10.

### Microscopic-macroscopic associated object pulling strategy.

A set of unlabeled microscopic images is classified as target domain *T* including T. gondii and host cells; another set of its associated macroscopic object images is used as source domain *S*, such as bananas. It is expected that source data share the same cluster point with target T. gondii images and that host cell images belong to another cluster point. Then, we calculated the degree of membership of each cluster point for each sample in *T*. Furthermore, an image translator is used to pull the target T. gondii images closer to source banana images and the host cell images away from banana, which is achieved by replacing the domain label with the degrees of membership of each cluster point. Through the whole process, labeling of *T* is not required. We integrated Cycle GAN into the image translator combined with a fuzzy C-means algorithm to enhance its selectivity, by which discriminator labels are replaced by degrees of membership obtained in fuzzy C-means.

For a set of unannotated target domain *T* and its desired associated source domain *S*, Cycle GAN learns two mappings without any supervision, which are *G*:*S*→*T* and *F*:*T*→*S* with two generators *G* and *F*. To bypass the infeasibility of pixel-wise reconstruction with unpaired data, i.e., *G*(*S*) ≈ *T* or *F*(*T*) ≈ *S*, Cycle GAN introduces an effective cycle consistency loss for *F*(*G*(*S*)) ≈ *S* and *G*(*F*(*T*)) ≈ *T*. The idea is that the generated target domain data are able to return to the exact data in the source domain from which they are generated. To guarantee the fidelity of fake data *G*(*S*) and *F*(*T*), Cycle GAN uses two discriminators *D_S_* and *D_T_* to distinguish real or synthetic data and thereby encourage generators to synthesize realistic data.

To solve the task of learning generators with unpaired images from two domains, *S* and *T*, we adopted the idea of the original cycle consistency loss (10) for generators *G* and *F*, forcing the reconstructed synthetic samples *F*(*G*(*x_S_*)) and *G*(*F*(*x_T_*)) to be identical to their inputs *x_S_* and *x_T_*.

(2) $$\begin{array}{ll} L_{\text{cyc}}(G,F) & =E_{x_{S}∼P_{\text{data}}(S)}[||F(G(x_{S}))-x_{S}|{|}_{1}]\\ & +E_{x_{T}∼P_{\text{data}}(T)}[||G(F(x_{T}))-x_{T}|{|}_{1}] \end{array}$$

where *x_S_* is the image from source domain *S* and *x_T_* is from target domain *T*. *L*_cyc_ uses the *L*_1_ loss, which shows better visual results than the *L*_2_ loss.

### Challenges in M2AOP strategy.

Lacking supervision with a direct reconstruction error between *G*(*S*) and *T* or *F*(*T*) and *S* brings some uncertainties and difficulties to the desired strengthened output of T. gondii and weakened output of host cells. The conventional Cycle GAN cannot evaluate the importance of each sample, and it will transform all images in the *T* domain without any selectivity. That makes our task even more challenging.

Such a problem means that Cycle GAN cannot exploit the discriminative information from the associated object in *S* and cannot select effective target samples from *T* in an unsupervised manner. Our goal is to pull target T. gondii images closer to banana images in *S* and make the other host cell images remain a great distance from source banana images. So, we designed the FCGAN approach, consisting of source target image translated consistency, a fuzzy pulling cluster generator, a discriminator, and its optimization.

### Fuzzy cycle generative adversarial network.

Aiming to obtain selectivity on Cycle GAN in T. gondii recognition without the help of microscopic annotations, we considered using a cluster algorithm. Specifically, a fuzzy clustering algorithm resolves this dilemma by introducing a degree of membership for each data point belonging to an arbitrary number of clusters (12).

We embedded the degree of membership into Cycle GAN, which means the degree of each sample belonging to the T. gondii cluster point and host cell point. We assumed that T. gondii shares a cluster point with banana images in *S*; then, the cluster closer to the source banana point is the T. gondii cluster, and the other point represents host cell images. Then, we developed the generator loss especially for discriminators embedded with degrees of membership into adversarial loss. It could strengthen the generativity of embedded Cycle GAN for samples belonging to the *T. gondii* group and weaken the samples belonging to the host cell group. As a result, the cluster points of T. gondii and the host cell can be separated with a greater distance in the embedded Cycle GAN.

For this idea, we introduced the fuzzy C-means (FCM) algorithm (13). To learn representative features, we designed a feature extractor *C* improved from the visual geometry group (VGG) network (11). For images {*x*_1_, …, *x_k_*, …, *x_N_*} in target domain *T* (their translated fake source images are {*F*(*x*_1_), …, *F*(*x_k_*), …, *F*(*x_N_*)}), we can get their feature space after translation of {*C*(*F*(*x*_1_)), …, *C*(*F*(*x_k_*)), …, *C*(*F*(*x_N_*))} through the feature extractor *C*. Partition of these features into *P* clusters with minimization is defined as the following objective function:

(3) $$L_{\text{fuzzy}}=\sum_{i=1}^{P}\sum_{k=1}^{N}u_{\mathrm{ik}}^{m}||C(F(x_{k}))-v_{i}|{|}^{2}$$

where *x_k_* is the *k*th image in domain *T*; *v_i_* is the *d*-dimensional center of the *i*th cluster; *u_ik_* denotes the degree of membership of feature *C*(*F*(*x_k_*)) to cluster *v_i_*; *N* is the total number of features in each domain; and ‖ *C*(*F*(*x_k_*)) − *v_i_* ‖ is any norm expressing the similarity between the measured feature *C*(*F*(*x_k_*)) and the cluster center *v_i_*. *m* denotes a weighting exponent parameter (*m *> 1) on each fuzzy membership value, and it determines the amount of fuzziness of the resulting classification according to:

(4) $$u_{\mathrm{ik}}\in [0,1];\sum_{i=1}^{P}u_{\mathrm{ik}}=1,\forall k;0<\sum_{k=1}^{N}u_{\mathrm{ik}}<N,\forall i$$

The first main step of this iterative algorithm is to update the membership function to determine in which cluster the feature belongs according to:

(5) $$u_{\mathrm{ik}}=\frac{1}{\sum_{j=1}^{C}{\left(\frac{\left||v_{i}-C(F(x_{k}))|\right|}{\left||v_{j}-C(F(x_{k}))|\right|}\right)}^{\frac{2}{\left(m-1\right)}}}$$

where *C* is a feature extractor.

The second main step concerns the updated centroids based on the new membership matrix according to:

(6) $$v_{i}(x)=\frac{\sum_{k=1}^{N}{\left(u_{\mathrm{ik}}\right)}^{m}C(F(x_{k}))}{\sum_{k=1}^{N}{\left(u_{\mathrm{ik}}\right)}^{m}}$$

Finally, we computed the objective function related to equation 3 and checked the criterion termination (the objective function convergence).

The fuzzy membership matrix *U* consists of the degree of membership belonging to each cluster for each sample feature. We embedded *U*, which has two degrees belonging to T. gondii and host cell for each feature, denoted as *U* = {*U_S_*, *U_T_*}, into our Cycle GAN and designed the fuzzy Cycle GAN loss for generators {*G*, *F*} and discriminators {*D_S_*, *D_T_*}. Their corresponding loss constraints are:

(7) $$\{\begin{array}{ll} L_{F}=E_{x_{T}∼p_{\text{data}(T)}}[\text{log}(U_{S}-D_{S}(F(x_{T})))], & \\ L_{D_{S}}=E_{x_{S}∼p_{\text{data}(S)}}[\text{log}D_{S}(x_{S})] & \\ \;+E_{x_{T}∼p_{\text{data}(T)}}[\text{log}(U_{T}-D_{S}(F(x_{T})))], & \end{array}$$

(8) $$\{\begin{array}{ll} L_{G}=E_{x_{S}∼p_{\text{data}(S)}}[\text{log}(1-D_{T}(G(x_{S})))], & \\ L_{D_{T}}=E_{x_{T}∼p_{\text{data}(T)}}[\text{log}D_{T}(x_{T})] & \end{array}$$

where *U_S_* is the degrees of membership belonging to the shared cluster point of source banana images in *S* and T. gondii images in *T*, and *U_T_* is the degrees of membership belonging to the host cell cluster point in *T*. From these two constraints, *U_S_*(*U_T_*) instead of label 1 in *D_S_*(*D_T_*) makes fake images from T. gondii images more similar to images in *S* than host cell images.

**FIG 7:**
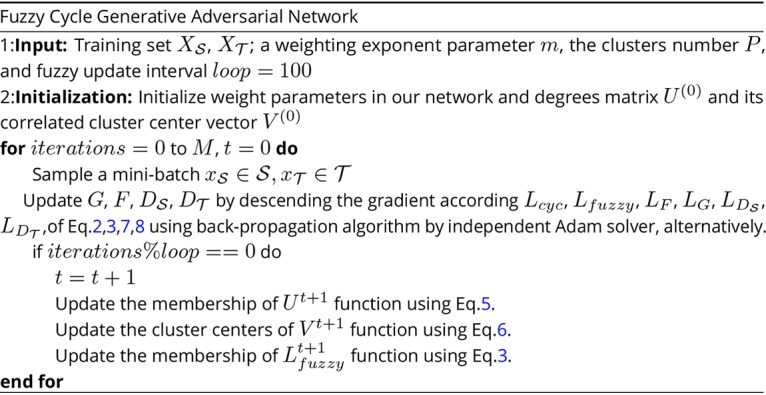
The algorithm of the fuzzy cycle generative adversarial network (FCGAN).

Figure 7 summarizes the main step of this sequential iterative algorithm with back-propagation and the Lagrange multiplier method.

### Training details.

Our FCGAN approach was designed from cycle GAN and VGG layers to conduct the algorithm, while we made several critical modifications to them. First, using the VGG network (11) to learn feature representation is critical to maintain the discriminative information for microscopic images, as it has achieved much faster convergence and locally smooth results in malaria recognition (8). Second, we adopted the degrees of membership into the generative adversarial loss function to enhance the generativity of sample group belonging to the closed cluster between source domains and weaken the sample group belonging to the distant cluster between source domains for generators and discriminators. Such an approach can separate the different groups with a great distance.

We implemented our network on the TensorFlow framework (14) with Tesla K40C and 128G memory in an Ubuntu 16.04 system and used the Adam solver (15) for FCGAN with a learning rate of 2 × 10^−4^ for generators and 2 × 10^−6^ for feature extractor, closely following the settings of Cycle GAN and VGG network to train generators, discriminators, and feature extractors. After jointly training for 4,000 iterations, we applied an early stop when the fuzzy loss no longer decreased for about 5 iterations (it usually takes 4,000 joint training iterations to reach a desired point). In training, the number of training data in two domains can be different. Our parameters in the algorithm are set as *m *= 2, *P* = 2, and *loop* = 100.

### Image collection and evaluation.

The stained T. gondii microscopic images were captured under two bright-field light microscopes (Leica DM2700P and Olympus IX53) in China, where preserved slices of parasite infection samples were mounted onto slides and stained with Giemsa stain. The first data set was captured with ×40 objectives (the magnification was ×400 [T400]) in the Leica DM2700P microscope, obtaining 8,156 T. gondii images and 4,979 host cell images. The second data set was captured with ×100 oil immersion objectives (the magnification was ×1,000 [T1000]) in the Olympus IX53 microscope, which obtained 6,969 T. gondii images and 8,023 host cell images. Moreover, 500 unstained T. gondii images and 500 host cell images were captured in Colombia by an EVOS FL Auto cell imaging system with ×400 magnification. In addition, we compiled 2,382 banana images, 2,053 crescent images, and 1,860 star images from the Internet as different source data to compare macroscopic associated objects.

Notably, different-magnification microscope slides typically display variations in color, background, and illumination between slides from different technicians, laboratories, clinics, and regions. Color variation can result from differences in staining pH, time and purity of dye, duration of the staining procedure, and the sensor settings (Fig. 1). If uncorrected, these variations may degrade the model performance. To overcome negative effects of variations, we adopted white balancing techniques to compensate for some of these variations. For all images, we used the white balancing technique to pool the pixels from all fields of view and computed a global color balance affine transform for each image. Then, we used a level-set-based algorithm (16) to crop T. gondii and host cells, resize all images in 256 × 256, and normalize each pixel in [0,1] by (*x* − μ)/σ before feeding into the deep learning network, where *x* denotes each pixel in images and μ = σ = 127.5 in our setting.

To evaluate the overall recognition performance, we calculated the average recognition accuracy for our parasite data sets and computed the F1 score, recall, and precision for parasites and their host cells. The average values are summarized in Table 1. We also performed average area under the receiver operating characteristic (ROC) curves for binary classification (17), and the ROC curves are drawn in Fig. 2. AUC measures the probability that a given pair of samples will have different class labels. If a sample is from class *c*, the classifier will be assigned a high prediction score, compared to the samples from other classes. Here, the prediction score is calculated by its degree of membership.

To validate the effectiveness of our FCGAN approach for the T. gondii recognition task, we first compared FCGAN with three supervised deep convolutional neural networks (CNNs), including deep residual network (ResNet) (18), visual geometry group network (VggNet) (11), and Google Inception v4 network (GoogleNet) (19). We selected half-samples to train each supervised network and test on the left images for two data sets. Besides, to discuss effects of components in our FCGAN, we implemented some unsupervised experiments to evaluate effects of each component in FCGAN. VggFCM (ResFCM, GoogleFCM) is a combination of the VGG (Res, Google) network with the fuzzy C-means (FCM) cluster algorithm, and CycleFCM combines the VGG+FCM and conventional cycle generative adversarial network (Cycle GAN), which can prove the efficiency of the embedded fuzzy component in Cycle GAN. Then, we also replaced Cycle GAN with a generative adversarial network (GAN) (20) and produced two baselines as fuzzyGAN with fuzzy domain labels, GanFCM with conventional GAN and FCM. Furthermore, we chose stars and crescents as another source of macroscopic objects besides bananas, because a star is not similar but a crescent is close to T. gondii.

To prove the main idea that the more similar the macroscopic object, the better the performance that will be achieved in the FCGAN, we also performed extensive evaluations, including (i) cross-domain image generation to test the fuzzy component, (ii) feature map visualization for convolutional layers, and (iii) t-SNE plots for feature extraction.

### Data availability.

The data and codes that support the findings of this study are available on GitHub at https://github.com/senli2018/FCGAN/.

## References

[1] Khan A, Grigg ME 2017 Toxoplasma gondii: laboratory maintenance and growth. Curr Protoc Microbiol 44:20C.1.1–20C.1.17. doi:10.1002/cpmc.26. PMC5537724 28166387

[2] Burrells A, Taroda A, Opsteegh M, Schares G, Benavides J, Dam-Deisz C, Bartley PM, Chianini F, Villena I, van der Giessen J, Innes EA, Katzer F 2018 Detection and dissemination of Toxoplasma gondii in experimentally infected calves, a single test does not tell the whole story. Parasit Vectors 11:45. doi:10.1186/s13071-018-2632-z. 29347971 PMC5774111

[3] Christiansen EM, Yang SJ, Ando DM, Javaherian A, Skibinski G, Lipnick S, Mount E, O’Neil A, Shah K, Lee AK, Goyal P, Fedus W, Poplin R, Esteva A, Berndl M, Rubin LL, Nelson P, Finkbeiner S 2018 In silico labeling: predicting fluorescent labels in unlabeled images. Cell 173:792–803. doi:10.1016/j.cell.2018.03.040. 29656897 PMC6309178

[4] Loddo A, Di Ruberto C, Kocher M 2018 Recent advances of malaria parasites detection systems based on mathematical morphology. Sensors 18:E513. doi:10.3390/s18020513. 29419781 PMC5856187

[5] Gecer B, Aksoy S, Mercan E, Shapiro LG, Weaver DL, Elmore JG 2018 Detection and classification of cancer in whole slide breast histopathology images using deep convolutional networks. Pattern Recognit 84:345–356. doi:10.1016/j.patcog.2018.07.022. 30679879 PMC6342566

[6] Xing F, Xie Y, Su H, Liu F, Yang L 2018 Deep learning in microscopy image analysis: a survey. IEEE Trans Neural Netw Learn Syst 29:4550–4568. doi:10.1109/TNNLS.2017.2766168. 29989994

[7] Rajaraman S, Antani SK, Poostchi M, Silamut K, Hossain MA, Maude RJ, Jaeger S, Thoma GR 2018 Pre-trained convolutional neural networks as feature extractors toward improved malaria parasite detection in thin blood smear images. PeerJ 6:e4568. doi:10.7717/peerj.4568. 29682411 PMC5907772

[8] Mehanian C, Jaiswal M, Delahunt C, Thompson C, Horning M, Hu L, Ostbye T, McGuire S, Mehanian M, Champlin C 2017 Computer-automated malaria diagnosis and quantitation using convolutional neural networks, p 116–125. *In* Proceedings of the 2017 IEEE International Conference on Computer Vision Workshops.

[9] Bezdek JC 1973 Cluster validity with fuzzy sets. J Cybern 3:58–73. doi:10.1080/01969727308546047.

[10] Zhu JY, Park T, Isola P, Efros AA 2017 Unpaired image-to-image translation using cycle-consistent adversarial networks, p 2223–2232. *In* Proceedings of the IEEE International Conference on Computer Vision Workshops.

[11] Simonyan K, Zisserman A 2015 Very deep convolutional networks for large-scale image recognition. *In* Bengio Y, LeCun Y (ed), Proceedings of the 3rd International Conference on Learning Representations.

[12] Simon D 1996 Fuzzy sets and fuzzy logic: theory and applications. Control Eng Pract 4:1332–1333. doi:10.1016/0967-0661(96)81492-4.

[13] Bezdek JC 1973 Fuzzy mathematics in pattern classification. Ph.D. dissertation. Cornell University, Ithaca, NY.

[14] Abadi M, Barham P, Chen J, Chen Z, Davis A, Dean J, Devin M, Ghemawat S, Irving G, Isard M, Kudlur M, Levenberg J, Monga R, Moore S, Murray DG, Steiner B, Tucker PA, Vasudevan V, Warden P, Wicke M, Yu Y, Zheng X 2016 TensorFlow: a system for large-scale machine learning, p 265–283. *In* Proceedings of the 12th USENIX Symposium on Operating Systems Design and Implementation.

[15] Kingma DP, Ba J 2015 Adam: a method for stochastic optimization. *In* Proceedings of the 3rd International Conference for Learning Representations.

[16] Lankton S, Tannenbaum AR 2008 Localizing region-based active contours. IEEE Trans Image Process 17:2029–2039. doi:10.1109/TIP.2008.2004611. 18854247 PMC2796112

[17] Sirinukunwattana K, Raza S, Tsang YW, Snead D, Cree I, Rajpoot N 2016 Locality sensitive deep learning for detection and classification of nuclei in routine colon cancer histology images. IEEE Trans Med Imaging 35:1196–1206. doi:10.1109/TMI.2016.2525803. 26863654

[18] He K, Zhang X, Ren S, Sun J 2015 Deep residual learning for image recognition. *In* Proceedings of the IEEE Conference on Computer Vision and Pattern Recognition.

[19] Szegedy C, Ioffe S, Vanhoucke V, Alemi AA 2017 Inception-v4, Inception-ResNet and the impact of residual connections on learning. *In* Proceedings of the Thirty-First AAAI Conference on Artificial Intelligence.

[20] Yu Y, Gong Z, Zhong P, Shan J 2017 Unsupervised representation learning with deep convolutional neural network for remote sensing images, p 97–108. *In* Zhao Y, Kong X, Taubman D (ed), Image and graphics. ICIG 2017 Springer, Cham, Switzerland.

